# The dual role and therapeutic potential of high-mobility group box 1 in cancer

**DOI:** 10.18632/oncotarget.17885

**Published:** 2017-05-16

**Authors:** Si-Jia He, Jin Cheng, Xiao Feng, Yang Yu, Ling Tian, Qian Huang

**Affiliations:** ^1^ Cancer Center, Shanghai General Hospital, Shanghai Jiao Tong University School of Medicine, Shanghai, China; ^2^ Shanghai Key Laboratory of Pancreatic Diseases, Shanghai General Hospital, Shanghai Jiao Tong University School of Medicine, Shanghai, China; ^3^ Oncology Department, Henan Provincial People's Hospital, Zhengzhou, China; ^4^ Institute of Translational Medicine, Shanghai General Hospital, Shanghai Jiao Tong University School of Medicine, Shanghai, China

**Keywords:** HMGB1, RAGE, TLRs, cancer, anticancer therapy

## Abstract

High-mobility group box 1 (HMGB1) is an abundant protein in most eukaryocytes. It can bind to several receptors such as advanced glycation end products (RAGE) and Toll-like receptors (TLRs), in direct or indirect way. The biological effects of HMGB1 depend on its expression and subcellular location. Inside the nucleus, HMGB1 is engaged in many DNA events such as DNA repair, transcription, telomere maintenance, and genome stability. While outside the nucleus, it possesses more complicated functions, including regulating cell proliferation, autophagy, inflammation and immunity. During tumor development, HMGB1 has been characterized as both a pro- and anti-tumoral protein by either promoting or suppressing tumor growth, proliferation, angiogenesis, invasion and metastasis. However, the current knowledge concerning the positive and negative effects of HMGB1 on tumor development is not explicit. Here, we evaluate the role of HMGB1 in tumor development and attempt to reconcile the dual effects of HMGB1 in carcinogenesis. Furthermore, we would like to present current strategies targeting against HMGB1, its receptor or release, which have shown potentially therapeutic value in cancer intervention.

## INTRODUCTION

High-mobility group box 1 protein (HMGB1), also known as amphoterin or HMG1, is an evolutionarily conserved non-histone DNA-binding protein and named for its high electrophoretic mobility on polyacrylamide gels. It was first identified as a group of chromatin-associated protein with high acidic and basic amino acid contents in 1973 [1]. HMGB1 is widely expressed in most mammalian cells, except those erythrocytes and cornifying epithelial cells that have eliminated nucleus. Nuclear HMGB1 is engaged in many DNA activities, such as DNA stability, repair, transcription and recombination. While in extracellular space, HMGB1 plays a more complicated role. It acts as the prototypic damage-associated molecular pattern molecule (DAMP) and interacts with several receptors including the receptor for advanced glycation end products (RAGE), Toll-like receptors (TLRs) and others for diverse biological functions. Since its discovery, HMGB1 has been implicated in many disease states including inflammation, immune disorders and cancer. In cancer, the dysfunction of HMGB1 has been verified to be associated with all the central hallmarks of cancer [2]. This review is focused on the current knowledge of HMGB1 biological features, especially in relation to the development of cancer and its potential therapeutic values.

## THE BIOLOGY OF HMGB1

### Structure

**Figure 1:**
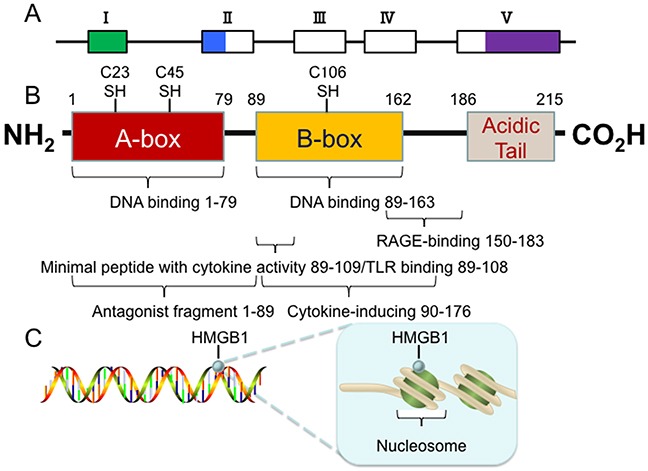
Structure of the HMGB1 protein **(A)** The 5 exons of human HMGB1 gene are indicated by boxes (hollow for translated regions and solid for un-translated regions). **(B)** The human HMGB1 protein has 215 amino acid residues and is composed of three domains: A box, B box and an acidic C-terminal tail. There are three redox-sensitive cysteine residues at positions 23, 45, and 106, which regulate HMGB1 function in response to oxidative stress. **(C)** The human HMGB1 is loosely and transiently associated with nucleosomes. In this location, HMGB1 is important for spatial segregation and nuclear homeostasis.

The high-mobility group box (HMGB) protein family is a group of highly conserved protein family that contains HMG box domains. HMG box is a novel type of DNA binding motif that is characterized by three α-helices arranged in an L-shaped configuration [3]. There are four members in this group: HMGB1, HMGB2, HMGB3 and HMGB4. HMGB1, encoded on human chromosome 13q12-13, is a relatively small protein with three distinct domains: two tandem HMG box domains (A box and B box) and an acidic C-terminal tail, containing a stretch of approximately 30 continuous glutamic and aspartic acid residues [3] (Figure 1). Functionally, the A (1-79 amino acid) and B (89-162 amino acid) boxes can interact with DNA to bend or distort the double helix [4]. The B box recapitulates the cytokine activity of full length HMGB1 and efficiently induces macrophage secretion of additional pro-inflammatory cytokines [5]. This cytokine activity can be antagonized by recombinant A box [6]. Residues 150-183 are responsible for binding with receptor for advanced glycation end products (RAGE), whereas residues 89-108 are responsible for binding with Toll-like receptors (TLRs) [4]. The C-terminal tail (186-215 amino acid) contributes to the spatial arrangement of both A and B boxes and modulates HMGB1 DNA-binding specificity [7]. It folds back to the N-terminal part of HMGB1, serving as a lid to cover the DNA binding domains, which only allows the DNA with special structure to assess the DNA binding domains [8]. This feature makes HMGB1 not occupied by linear DNAs in the nucleus [8]. Removal of the C-tail from HMGB1 enhances its DNA-binding activity, but weakens its preference for bended DNAs over linear DNAs [8]. Importantly, there are three redox-sensitive cysteine residues at positions 23, 45, and 106, which are highly conserved across all the species [4]. Cysteine 23 and cysteine 45 are located within the A box and can form an intramolecular disulfide bond in response to oxidative stress, which is required for binding to Beclin1 and sustaining autophagy [9]. However, cysteine 106, which is unpaired and located within the B box, appears to be critical for the nucleocytoplasmic shuttling of HMGB1 [2, 10].

### Receptor

There are receptors that directly bind to HMGB1, such as RAGE, TLR2, TLR4 and TIM3. However, there are several receptors associated with HMGB1, including TLR9, CXCR4. All these receptors with or without direct binding to HMGB1 exert diverse pathological and physiological functions.

#### RAGE

Receptor for advanced glycation end products (RAGE) is a transmembrane receptor encoded by gene on chromosome 6p21.3. The extracellular domain of RAGE, which is used for ligand binding, contains one variable-like (V) and two constant-like (C) type domains. The V domain has two N-glycosylation sites and is responsible for extracellular ligand binding. The 43-amino acid cytoplasmic tail of RAGE is required for intracellular signaling transduction. RAGE has several isoforms deriving from alternative splicing including full-length (fl-RAGE), membrane-bound (mRAGE) and various soluble forms lacking the transmembrane domain [11]. Soluble RAGE can be generated through proteolytic cleavage of mRAGE by the sheddase called a disintegrin and metalloprotease 10 (ADAM10). The sheddase ADAM10 is a membrane protease, which promotes RAGE shedding via binding its ligand HMGB1 [12]. RAGE is now known as a multi-ligand receptor and binds to a number of ligands such as advanced glycation end products (AGE), HMGB1, β-amyloid fibrils, certain S100 proteins, DNA, RNA and other molecules, adjusting a series of physiological activities and pathological processes [13, 14]. A great number of studies demonstrate that RAGE is indispensable for HMGB1-induced cell proliferation, regeneration, migration, inflammation, autophagy and immunity [14–16]. Knockdown of RAGE by specific siRNA or RAGE ligand application increased cancer cell proliferation [15]. Knockout of RAGE decreases tumor growth and metastasis, increases chemotherapy resistance both *in vitro* and *in vivo* [17]. It is demonstrated that the HMGB1-RAGE signaling axis contributes to cell proliferation and metastasis by inducing nuclear factor κB (NF-κB) activation, revealing HMGB1-RAGE as a potential target for therapeutic intervention in cancer [18].

#### TLRs

The Toll-like receptors (TLRs) are a family of transmembrane receptors that contain extracellular leucine-rich repeats (LRRs) and a cytoplasmic Toll/interleukin-1 receptor (TIR) domain. The LRRs are used for ligand binding and trigger signal transduction pathways through TIR domains with conserved adaptor molecules [19]. There are five adaptor molecules that link TLRs to downstream kinases: myeloid differentiation primary response gene 88 (MyD88), TIR domain-containing adaptor protein (TIRAP, also known as Mal), TIR domain-containing adaptor protein inducing IFN-β (TRIF, also known as TICAM1), TRIF-related adaptor molecule (TRAM, also known as TICAM2) and sterile α and HEAT-Armadillo motifs (SARM) [20]. Most TLRs signal utilizes MyD88, except that TLR3 signal merely utilizes TRIF. TLR4 is the only receptor that transduces signals through MyD88 and TRIF. TLR signaling pathways are broadly classified as the MyD88-dependent and MyD88-independent pathways. After binding with individual ligands, TLRs recruit MyD88 or other adaptor molecules, leading to multiple activation of downstream factors, such as NF-κB, mitogen-associated protein kinase (MAPK), and interferon (IFN) regulatory factors [19]. TLRs are mainly expressed in innate immune cells, inducing and regulating adaptive immune responses. However, TLRs are also expressed on other cell types, including endothelial, epithelial and tumor cells. Modulation of TLR signaling can confer anti- or pro-tumor effects, which depends on the TLR, cancer subtype and the tumor-infiltrating immune cells [21]. The anti-tumor effects can be achieved by inducing direct tumor cell death or improving anti-tumor immune responses, and the pro-tumor effects can result from activating tumor cell survival and proliferation signaling pathways or suppressing inflammatory immune cells in the tumor microenvironment [21]. TLRs are pattern recognition receptors (PRRs) that recognize a wide range of pathogen-associated molecular patterns (PAMPs) and endogenous damage-associated molecular patterns (DAMPs). As such, HMGB1 can interact with TLRs and then activate relevant signal transduction pathways to produce a series of cytokines and chemokines. It is demonstrated that irradiated tumor cells released HMGB1 can activate TLR4 on dendritic cells and lead to tumor elimination by tumor-specific T cells [22]. Knockout of TLR4 decreases HMGB1-induced tissue injury, cell migration and adhesion, angiogenesis, inflammation and immunity responses [22, 23]. In addition, TLR2 is connected with tissue injury, cell migration and adhesion, inflammation, and stem cell self-renewal [24]. TLR9 is initially localized in the endoplasmic reticulum (ER), and redistributes to early endosomes upon stimulation with CpG-DNA via an HMGB1-dependent way [25]. Inhibition of both TLR9 and HMGB1 suppresses inflammatory cytokine secretion and confers protection from liver ischemia-reperfusion injury [26].

#### TIM3

T cell immunoglobulin domain and mucin domain-3 (TIM3) is a member of the TIM family consisting of an N-terminal immunoglobulin variable (IgV) domain followed by a mucin domain, a transmembrane domain and a cytoplasmic tail [27]. TIM3 is located on human chromosome 5q33.2 [28] and expressed on dendritic cells (DCs), monocytes, macrophages, and natural kill (NK) cells [29]. It is a marker for NK-cell activation or maturation and can suppress NK cell-mediated cytotoxicity when cross-linked [30]. The interaction between galectin9 and TIM3 can induce apoptosis in T cells [30]. DC-derived TIM3 can interact with HMGB1 and suppress the recruitment of nucleic acids to endosomes, which attenuates the antitumor efficacy of DNA vaccines and chemotherapy [31]. Blockade of TIM3 improves antitumor efficacy of anticancer cytotoxic agents by augmenting HMGB1-mediated nucleic acid-sensing systems [29, 30]. As TIM3 also serves as a receptor for phosphatidylserine (PtdSer) for the engulfment of apoptotic cells, blockade of TIM3 impairs the phagocytotic capacity of DCs, thus impeding the recognition of dying tumor cells [31].

#### CXCR4

C-X-C chemokine receptor type 4 (CXCR4) is a member of the G protein-coupled seven-transmembrane receptors (GPCRs), which is expressed lower in normal tissues but significantly higher in tumor tissues. CXCR4 overexpression in tumor tissue has been correlated to tumor aggressiveness, high risk of metastasis and recurrence [32]. CXCR4 is well known to be a co-receptor for CD4^<sup>+</sup>^ T-cell infection of human immunodeficiency virus (HIV) type 1 [33]. Chemokine stromal cell-derived factor-1 (SDF-1, also known as CXC chemokine ligand 12) is a ligand of CXCR4. The attraction between CXCR4 and SDF-1 promotes cell migration and invasion by activation of RAS/MAPKs, JAK/STAT and AKT/PI3K [34, 35]. Recent evidence demonstrates that the heterocomplex formed by HMGB1 and CXCL12 can bind to CXCR4 and promote recruitment of inflammatory cells to damaged tissues [36]. NF-κB signaling pathway is critical to sustain CXCL12/SDF-1 production for cells to migrate toward HMGB1, indicating that HMGB1-mediated cell migration is regulated through NF-κB signaling pathway [37].

### Expression and subcellular location

In general, HMGB1 is ubiquitously expressed to a very high level, which is believed to be only 10 times less than core histones. However, HMGB1 expression and subcellular localization varies depending on cell types, tissues and developmentally regulated to cues from the environment (Table 1) [38].

**Table 1:** A summary of data on HMGB1 expression and localization in normal and tumor tissue

Tissue	HMGB1 level	Subcellular location	Tumor	HMGB1 level	Subcellular location	Reference
Liver	Low	C	Hepatocellular carcinoma	High	N, C	[130]
Stomach	Low	nd	Gastric carcinoma	High	N	[131]
Colon	Low	nd	Colorectal carcinoma	High	N,C N	[132]
Pancreas	Low	nd	Pancreatic carcinoma	High	N, C	[133]
Breast	Low	nd	Breast cancer	High	N, C	[134]
Cervix	Low	nd	Cervical carcinoma	High	N, C	[135]
Brain	Undetectable in most cells in adult mouse brain, present during development	C	Glioma	High	C, N	[136]
Thymus	High in young rats, low in old rats	N, C	Thymic epithelial tumors	High	N, C	[137]
Lymphoid tissues	Low	N, C	Non-Hodgkin lymphoma	High	N	[138]

N: nuclear localization; C: cytoplasmic localization; nd: not determined.

The expression of HMGB1 is high in both nuclei and cytoplasm of lymphoid tissues and testis, whilst low in cytoplasm of hepatic tissues and brain, mainly in the cytosol [39]. In mouse testis, HMGB1 appears to be expressed in spermatogonia, lower in spermatocytes, and absent in spermatids [38]. Immunohistochemistry of human arteries shows that HMGB1 is abundant in the nuclei of endothelial cells, but scarce in vascular smooth muscle cells [40]. The expression levels of HMGB1 also vary with age in rats, which is higher in the thymus of younger rats than the older rats [41]. The expression level of HMGB1 is associated with the differentiation stage of cells as well. Basically, HMGB1 expression is low in differentiated cells but high in undifferentiated cells [39]. The expression of HMGB1 in myeloid cells is higher than in lymphoid cells [42]. HMGB1 is highly expressed in most tumor types. Previous studies found that the expression of HMGB1 was much higher in tumor than in the normal tissue counterpart, such as hepatocellular carcinoma [15], breast carcinomas [43] and colorectal adenocarcinomas [44]. However, certain cancers such as adrenal gland carcinoma contain no HMGB1 protein [38].

Although HMGB1 is a nuclear protein, it can be transited and influenced by several posttranslational modifications such as acetylation, phosphorylation, methylation and oxidation. These modifications modulate HMGB1 structure, localization and subsequent biological functions [2], which will be discussed later.

### Release

#### Active release

**Figure 2:**
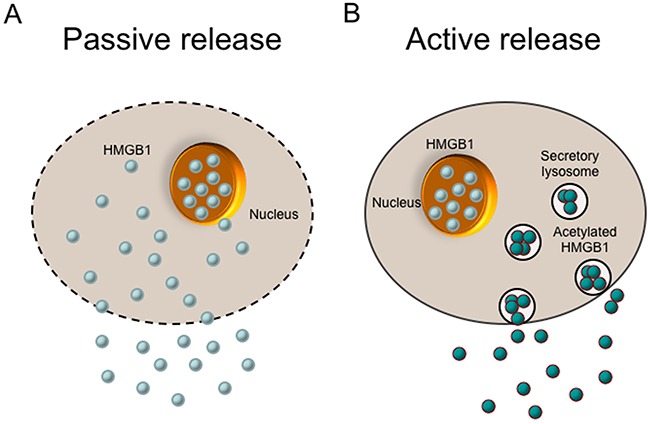
Release of the HMGB1 protein There are two mechanisms of cells to release HMGB1 into the extracellular environment (passive release vs. active release). **(A)** HMGB1 is passively released into extracellular space from damaged cells or necrotic cells with leaky plasma membranes. Apoptotic cells that undergo secondary necrosis can also motivate late HMGB1 release. **(B)** HMGB1 can be actively released from activated cells such as inflammatory cells and immune cells. In this process, HMGB1 is acetylated, which prevents it from getting back to nucleus. Then the cytoplasmic HMGB1 is enveloped into secretory lysosomes, fuses with the cell membrane, and finally released into the environment.

HMGB1 is a kind of ‘leaderless’ cytokine, which means it is not directly translocated from the Golgi apparatus to the cell surface. It requires access to be secreted in organelles that belong to the endolysosomal compartment [45]. The active release of HMGB1 initiates when extracellular molecules interact with cell membrane receptor or hypoxia occurs slowly. Many cell types can actively release HMGB1, such as monocytes, macrophages, DCs, NK cells, endothelial cells and tumor cells [46] (Figure 2). However, the mechanisms of this secretion may have discrepancies between different cell types. Monocytes have been the most deeply researched cell type in relation to secretion of HMGB1 so far. In monocytes, HMGB1 have to relocate from the nucleus to cytoplasmic secretory lysosomes or organelles, and then it can be released out of the cells, which is similar with the secretion of IL-1β. When monocytes activated by LPS, TNF, IL-1 or IFN-γ, HMGB1 will release out of the nucleus and accumulate in the cytoplasm. The cytoplasm HMGB1 is acetylated and phosphorylated so that it is forbidden to get into nuclear compartment again. Then the cytoplasmic HMGB1 is enveloped into secretory lysosomes and fuses with the cell membrane. The release of HMGB1 on cytoplasmic organelles is initiated by lysophosphatidylcholine [16]. Recently some studies have indicated that sublethal cellular hypoxia induces active HMGB1 release rather than passive release in a cell type-independent way. In synovitis, hypoxic areas were found to be coincided with areas of maximal pathological HMGB1 expression, which substantiated the connection between ischemia and HMGB1 translocation [26].

#### Passive release

The bond between HMGB1 and chromatin is loose, except that in granulocytes HMGB1 is tightly sequestered in an insoluble form. Thus, HMGB1 can be passively released into the extracellular space when the cells come through necrotic process (Figure 2). It should be noted that, in apoptotic cells, this does not occur, presumably on account that HMGB1 attaches to cruciform DNA or hypoacetylated proteins within apoptotic-cell nucleus [45]. However, apoptotic cells that may undergo secondary necrosis, motivating late HMGB1 release [46–48]. In mice rheumatoid arthritis model, HMGB1 can be secreted via necrotic macrophages and leaky apoptotic cells [47]. Furthermore, many of the macrophages that overtake undigested apoptotic bodies, get activated before cell death and actively secrete TNF, IL-1β, and possibly HMGB1 [46].

## THE FUNCTION OF HMGB1 IN CANCER

### Nuclear function of HMGB1

#### DNA chaperone

**Figure 3:**
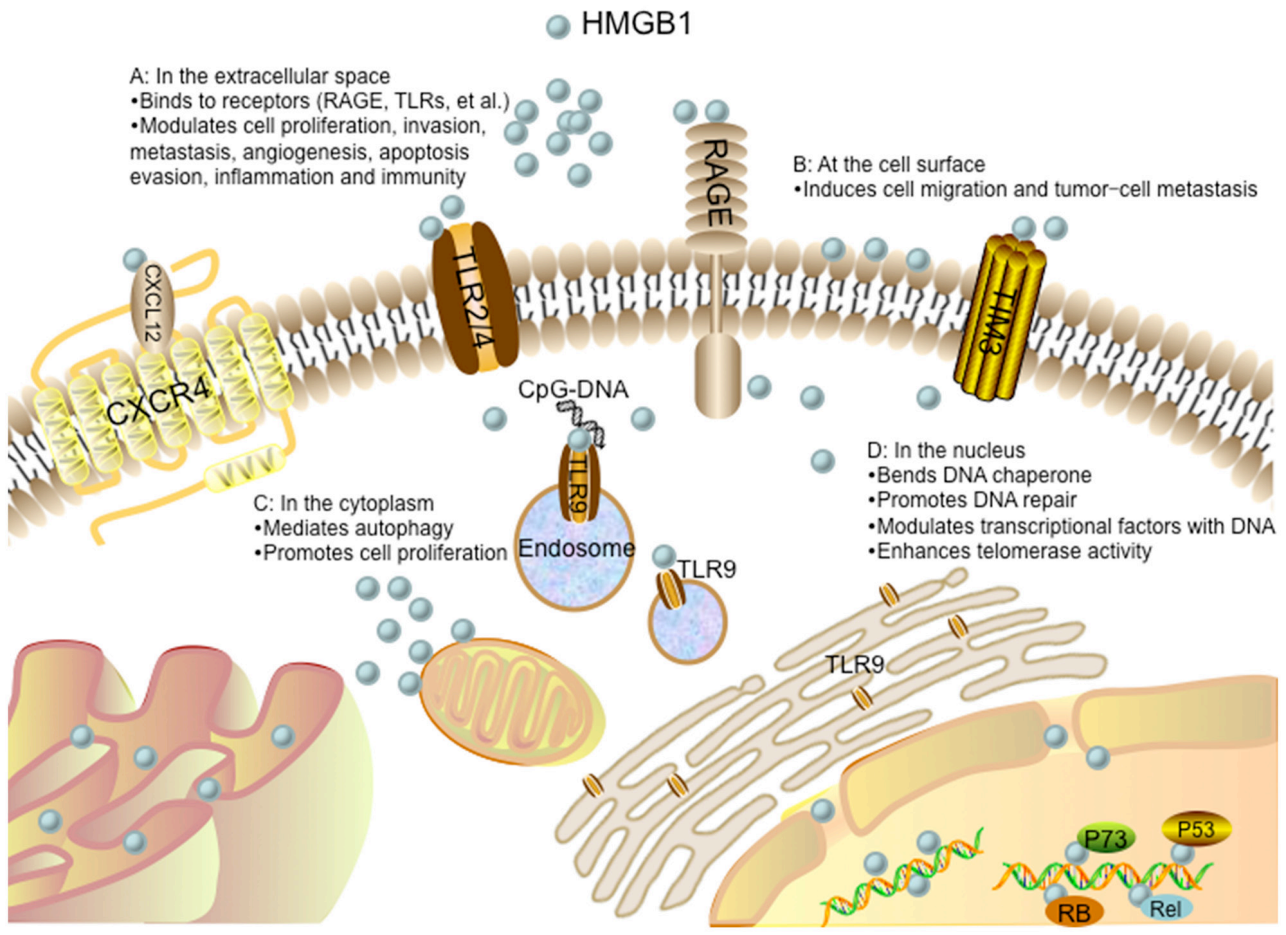
Role of the HMGB1 protein in cancer progression **(A)** In the extracellular space, HMGB1 signals through receptors (such as RAGE, TLRs, TIM3, and CXCR4), driving cell proliferation, invasion, metastasis, angiogenesis, apoptosis evasion, inflammation and immunity. The interaction between HMGB1 and CXCR4 is dependent on CXCL12. TLR9 is initially localized in the endoplasmic reticulum (ER), and redistributes to early endosomes upon stimulation with CpG-DNA via an HMGB1-dependent way. **(B)** HMGB1 present at the cell surface promotes cell migration and tumor-cell metastasis. **(C)** In the cytoplasm, HMGB1 regulates autophagy and promotes cell proliferation. **(D)** In the nucleus, HMGB1 acts as a DNA chaperone participating in DNA repair and transcription. HMGB1 can also interact with transcription factors and enhance their activities such as p53, p73, members of the Rel/NFκB family and RB. Nuclear HMGB1 enhances telomerase activity and modulates telomere homeostasis.

Nuclear HMGB1 acts as a DNA chaperone that binds to DNA structure without sequence-specificity (Figure 3). The flanking sequences of A box and B box as well as the acidic C terminal have the ability to regulate their DNA binding activities [49]. The binding between HMGB1 and DNA is enhanced when A box and B box are covalently connected. Post-translational modifications also regulate HMGB1 binding to DNA [50, 51]. For example, methylation of HMGB1 weakens its DNA binding activity by altering its conformation [52]. In addition, HMGB1 has a relatively high affinity to noncanonical DNA structures such as single-stranded DNA, synthetic cruciform structures, supercoiled DNA molecules and Z-DNA, preferentially DNA mini-circles, four-way junctions, looped structures and hemi-catenated DNA [49, 53]. Nuclear HMGB1 can also bend and change DNA conformation. HMGB1 binds to nucleosomes at the dyad axis and promotes nucleosome sliding, and bends to DNA to make chromatin more accessible [54]. The DNA bending activity of HMGB1 is maintained with HMG boxes [55]. The B box has “primary” intercalating Phe102 and “secondary” intercalating lle121, while the A box only has “secondary” intercalating Phe37. Thus, the B box contributes more to DNA bending. Mutation of these three intercalating residues prevent HMGB1 bind to chromatin and impair the ability of HMGB1 to bend DNA [49]. Generally, nuclear HMGB1 is identified as an “architectural” factor to facilitate the assembly of certain nucleoprotein complexes and participate in fundamental events such as DNA replication, remodeling and DNA repair [49].

#### DNA repair

Loss of HMGB1 or increased HMGB1 translocation from the nucleus to the cytoplasm increases DNA damage, decreases DNA repair efficiency, and intensifies cell response to various stimuli such as chemotherapy, irradiation and oxidative stress (Figure 3). There are two hypotheses of HMGB1 on the repair of damaged DNA. One is the “repair shielding” hypothesis, proposing that the binding of HMGB1 to the DNA lesion can block the access of the repair machinery [56]. The other is “repair enhancement” hypothesis, deducing that HMGB1 can bind and bend damaged DNA cooperatively with excision repair damage recognition proteins [57]. It has been shown that HMGB1 participated in all four major DNA repair pathways: mismatch repair (MMR) [58], base excision repair (BER) [59], double-strand break repair (DSBR) [60] and nucleotide excision repair (NER) [61]. HMGB1 bond to damaged DNA, altered DNA structures, interacted with repair enzymes or cofactors, and remodeled chromatin. Thus, dubbed HMGB1 the “jack-of-all-trades” repair protein [56]. Early researches found that HMGB1 specifically bond to damaged DNA isolated from cells treated with cisplatin [62, 63]. Cispaltin induces 1,2-intrastrand d (GpG) and d (ApG) cross-links in DNA. To repair this DNA lesion, NER, one of the cellular defense mechanisms against the toxic effects of cisplatin, is involved that links HMGB1 to this repair pathway [64]. Non-homologous end-joining (NHEJ) is the main DSBR pathway to ligate the damaged DNA ends of DSBs, which HMGB1 is believed to participate in several steps of NHEJ repair pathway. Purified HMGB1 has been reported to bind to the ends of DSBs and stimulate the activity of DNA-dependent protein kinase *in vitro* [65]. However, whether HMGB1 plays similar role in NHEJ repair of DSBs *in vivo* has been seldom studied. HMGB1 is identified as a participant in MMR as well. It is involved in the initial damage recognition/incision steps of heteroduplex repair and interaction with the MMR proteins MSH2 and MLH1 [58]. HMGB1 is confirmed to be responsible for cisplatin resistance in esophageal squamous cell carcinoma (ESCC) [66]. HMGB1 contributes to the repair of DNA damage and cells, lacking HMGB1, are hypersensitive to DNA damage precipitating factors [57].

#### Transcription

The role of HMGB1 in gene transcription is either an activator or a repressor (Figure 3). Generally, HMGB1 can bend DNA and facilitate the binding of transcription factors to their cognate DNA sequences and enhance their activities, such as p53 [67], p73 [68], estrogen receptor (ER) [69], replication and transcription activator (RTA) [70], the retinoblastoma protein (RB) [71], Rel/NF-κB [72]. P53 is the most frequently altered gene in human carcinoma. Previous studies have implicated that p53 carboxyl terminus negatively regulates its binding to DNA. Carboxy-terminally deleted p53 is observed to be constitutively active for DNA binding. However, the regulation of HMGB1 on p53 DNA binding is independent from that involving p53 carboxyl terminus. It is reported that HMGB1 induced DNA bending can provide prebent consensus site DNA to p53 and increase p53 activity [73]. The bent DNA can also recruit additional transcription factors in close vicinity and facilitate their mutual functions [49]. Moreover, HMGB1 can enhance DNA flexibility by looping and bring transcription factors bound on distant regulatory sequences into close proximity, which results in mutual contacts [49]. Additionally, it has been demonstrated that the HMGB1 can promote DNA binding of transcription activators without direct interactions between HMGB1 and transcription activators [74]. Besides, HMGB1 has been reported to act as a negative cofactor and repress transcription by RNA polymerase II. It binds to the TATA-binding protein (TBP) to form the HMGB1/TBP/TATA complex and prevents the recruitment of transcription factor IIB (TFIIB, a initiation factor) to this complex, subsequently inhibits the assembly of the pre-initiation complex (PIC) [75].

#### Telomere and telomerase

Telomeres are repetitive DNA sequences, consisting of two main core components: a catalytic protein subunit (telomerase reverse transcriptase, TERT) and an RNA subunit (telomerase RNA, TR) [76]. The telomere maintenance is believed to be a component of tumor cell unlimited replicative potential. HMGB1 is reported to modulate cellular activity of mammalian telomerase (Figure 3). Knockout of the HMGB1 gene in mouse embryonic fibroblasts (MEFs) results in telomere dysfunction and reduced telomerase activity, while overexpression of HMGB1 enhances telomerase activity [76]. The maintenance and protection of telomeres are also modulated by shelterin, which is composed of protection of telomere 1 (POT1), telomeric-repeat-binding factor 1 (TRF1), TRF1-interacting protein 2 (TIN2), the TIN2 and POT1 interacting protein 1 (TPP1), telomeric-repeat-binding factor 2 (TRF2) and the transcriptional repressor/activator protein (RAP1). Through the interaction with telomerase, this shelterin can control the telomere length to maintain the stability of telomeres [77]. Knockdown of HMGB1 modulates telomere homeostasis by downregulating expressions of telomere-binding proteins TTP1 and TRF1, thereby initiating apoptosis and increasing radiosensitivity in human breast cancer cells [78].

### Cytosolic function of HMGB1

#### Autophagy

The main function of cytoplasmic HMGB1 is autophagy modulation (Figure 3). Autophagy is a lysosomal degradation process that clears long-lived or dysfunctional proteins and organelles for cell survival during periods of starvation or other micro-environmental stresses. Stimuli that enhance reactive oxygen species facilitate translocation of HMGB1 to cytoplasm and enhance autophagic flux. It is demonstrated that the dissociation of Bcl-2 from Beclin1 is an important mechanism in autophagy activation. Cytosolic HMGB1 can disrupt the interaction between Beclin1 and Bcl-2 by directly binding to Beclin-1 with its intramolecular disulfide bridge (C23/45) and sustain autophagy [9]. Cytoplasts (anucleate cells) have been created to assess the role of cytoplasmic HMGB1 in the setting of autophagy. HMGB1 KO MEF cytoplasts shows a lower level of LC3 punctae than HMGB1 wild-type MEF cytoplasts in response to starvation, indicating that cytoplasmic HMGB1 is required for starvation-stimulated autophagy [9]. A series of studies have confirmed that Beclin1 binds to Bcl-2 via its BH3 domain that can be modified by JNK or ERK signaling pathway. HMGB1 can increase transcriptional activities of JNK and ERK pathway in human myeloid leukemia cells, which results in phosphorylation of Bcl-2 and its binding to Beclin1 [79]. HMGB1 and p53, the most frequently mutated gene in human cancers, are both implicated in regulation of balance between autophagy and apoptosis [80]. The function of p53 in autophagy is controlled by its subcellular localization. Generally, cytoplasmic p53 represses autophagy, while nuclear p53 stimulates autophagy [80]. It is demonstrated that HMGB1 and p53 form a complex that regulates the cytoplasmic localization of the binding protein and subsequent levels of autophagy. P53^<sup>−/−</sup>^ HCT116 cells rapidly increase expression of cytosolic HMGB1 and associated autophagy in response to stress. Conversely, HMGB1^<sup>−/−</sup>^ MEFs increase cytosolic translocation of p53 and decrease autophagy in response to stress. Interestingly, it is reported that HMGB1 does not influence p53-dependent expression of damage-regulated autophagy modulator (DRAM) and unc-51-like 1 (ULK1) within the nucleus during autophagy. However, HMGB1 is required for induction of autophagy when p53 is knockout or pharmacological inhibited, suggesting HMGB1 regulates the cytoplasmic but not the nuclear function of p53 during autophagy [80, 81].

#### Cell proliferation

Human mitochondrial DNA (mtDNA) is a 16569 bp, maternally inherited, closed circular double-stranded DNA molecule. The content of mtDNA is precisely modulated by cellular physiological conditions and diverse internal or external microenvironments, such as hypoxia and steroid hormone stimulation [82]. Amounts of evidence have supported a critical role of mtDNA in a broad range of human cancers and contributing to cancer commencement and promotion at various stages of oncogenesis [82]. It is shown that cytoplasmic HMGB1 senses and binds to mtDNA via sensing the CpG rich motifs in mtDNA. During hypoxia, HMGB1 translocates from nucleus to the cytosol, binds to mtDNA released from damaged mitochondria, and subsequently activates TLR9 signaling pathway to promote tumor cell proliferation [83]. Loss of HMGB1 leads to a defect in TLR9 signaling pathway and decreases tumor cell proliferation, whereas addition of HMGB1 leads to the activation of TLR9 signaling pathway and promotes tumor cell proliferation [83] (Figure 3).

#### Secretion

Lee et al. identified 74 putative HMGB1-binding proteins localized exclusively in the extra-nuclear region, and 60 proteins bond HMGB1 both in the nucleus and extra-nuclear region. Many of these binding proteins are important in carcinogenesis, such as cell cycle progression (40S ribosomal protein S15a and serum albumin precursor), cell proliferation (40S ribosomal protein S15a, 40S ribosomal protein S4, X isoform, coffilin-1, nucleophosmin and protein arginine N-methyltransferase 5), angiogenesis (annexin 2, ATP synthase subunit beta, myosin heavy chain 9, serum albumin precursor and nucleolin) and anti-apoptosis (alpha actinin-4, coffilin-1, HSPA5 protein and nucleophosmin). Besides, there are nine HMGB1-binding proteins related to intracellular protein transport and secretion, including alpha actinin-4, nucleophosmin, myosin IC isoform a, protein-enabled homologue, splicing factor, arginine/serine-rich 1, Ras-related protein Rab-10, annexin A2 and dpy-30 homologue (DPY30) [44]. Most secreted proteins have a signal peptide that directs their translocation to ER and collectively form secretome, which is also known as the conventional secretory pathway [84]. However, there is no signal peptide in HMGB1, predicting that HMGB1 is secreted via the unconventional secretory pathway. Moreover, studies have proved that these nine HMGB1-binding proteins are associated with the unconventional secretory pathway. For instant, myosin IC isoform a and Ras-related protein Rab-10 regulate vesicular transport and involved in protein secretion. DPY30 is involved in endosomal transport. Annexin A2 functions in aggregation and membrane fusion of phospholipid-containing vesicles. In addition, cytosolic HMGB1 is relevant to the lysosome and co-localized with lysosomal protein LAMP1 by immunofluorescence confocal microscopy [44]. These findings indicate that the secretion of HMGB1 is via the unconventional secretory pathway, particularly via secretory lysosomes [44] (Figure 3).

### Membrane function of HMGB1

The functions of HMGB1 on cell surface include platelet activation [85], erythroid maturation [86] and innate immunity [87]. In human resting platelets, HMGB1 and its mRNA are localized in the cytoplasm. When platelets are activated, part of HMGB1 could export to the external surface of platelets. While in the platelet surface, HMGB1 could enhance the rate of plasminogen activation and promote the generation of surface-bound plasmin [85]. Studies have showed that HMGB1 expressed on the surface of erythroblasts and macrophages involves in erythroblast-macrophage contact to promote erythroid cells proliferation and terminal maturation [86]. To characterize the role of HMGB1 in the innate immunity of newborns, Ciucci et al utilize flow cytometry and western blot to analyze HMGB1 expression in human cord blood (CB) mononuclear cells and adult peripheral blood (PB) mononuclear cells [87]. The results show that HMGB1 is expressed on cell surface membranes of myeloid dendritic cell precursors and lymphocytes, which can be upregulated by pro-inflammatory stimuli and subsequently secreted into extracellular environment. These secreted HMGB1 enhance the immune response in CB via engagement of γδ T lymphocytes and myeloid dendritic cell precursors [87]. Additionally, it has been proposed that HMGB1 present at the cell surface promotes cell migration and tumor metastasis [45] (Figure 3).

### Extracellular function of HMGB1

Extracellular HMGB1 acts as a DAMP with cytokine and chemokine activities, leading to various responses, such as cell proliferation, invasion, metastasis, angiogenesis, inflammation and immunity (Figure 3).

#### Cell proliferation

Extracellular HMGB1 regulates cell proliferation in several cells via different mechanisms. It is reported that HMGB1 releases from human malignant mesothelioma cells and promotes proliferation of these cells via an autocrine circuit [88]. Exogenous HMGB1 induces mouse mesangial cell proliferation by promoting the cell cycle transition from G0/G1 to S phase [89]. Tumor cells require adenosine triphosphate (ATP) to support proliferation. Exogenous HMGB1 and RAGE coordinately contribute to tumor cell ATP production and subsequent cell proliferation in a time- and dose-dependent manner. It is also observed that endogenous HMGB1 increases mitochondrial RAGE expression, which is associated with tumor cell ATP production via MEK-ERK-MAPK pathway. Lack of RAGE or inhibition of HMGB1 reduces ATP production and slows pancreatic cancer cell growth [90]. However, this proliferative effect varies in different cell types. Chitanuwat et al. investigate that human recombinant HMGB1 can significantly induce the proliferation of human gingival fibroblasts, which is not obvious in human periodontal ligament fibroblasts [91].

#### Invasion and metastasis

The finding outlined in elucidating the role of extracellular HMGB1 in regulation of tumor cell invasion and metastasis is controversial. In some studies, the interaction of extracellular HMGB1 and RAGE induces NF-кB activation or MAPK signaling pathway to promote tumor cell invasion and metastasis [18, 92]. Extracellular HMGB1 activates TLR4 and RAGE signal pathways, subsequently the downstream of caspase-1, and then pro-inflammatory cytokines IL-1β and IL-18 are cleaved and released, which in turn promote HCC invasion and metastasis in hypoxia. Treatment with HMGB1 reinforces the invasion of HCC cells [93]. However, Zuo et al. demonstrate that knockdown of HMGB1 increases cancer cell migration, invasion and metastasis *in vitro* and *in vivo*, which can be inhibited by recombinant human HMGB1, indicating that the inhibitory effect on cell migration is dependent on the HMGB1 in the microenvironment. They also discovers that this inhibitory effect of extracellular HMGB1 is via suppressing phosphorylation, nuclear translocation and activation of cyclic AMP response element-binding protein (CREB), which further reduces neuron Wiskott–Aldrich syndrome protein (nWASP) expression [94].

#### Angiogenesis

Angiogenesis is essential for the progression of most solid tumors. The vessel formation of these tumors depends on pro- and anti-angiogenic factors in tumor microenvironments. Endothelial cells (ECs) play pivotal roles in tumor angiogenesis and provide attractive novel therapeutic targets. Gene expression profiling results of ECs isolated from freshly resected colorectal tumors, normal colon tissue and placenta showed that HMGB1 was a tumor angiogenesis gene [95]. *In vitro* and *in vivo* HMGB1 can stimulate the migratory and sprouting capacity of ECs, which can be attenuated by interference in HMGB1 expression. Antibodies of HMGB1 inhibit tumor angiogenesis in chicken embryo chorioallantoic membrane (CAM) model [96]. In human oral squamous cell carcinoma, HMGB1 promotes VEGF secretion via binding to RAGE. Down-regulation of RAGE abrogates this effect [97]. HMGB1 can also provoke the recruitment and activation of macrophages to produce angiogenic factors such as vascular endothelial growth factor (VEGF), tumor necrosis factor-α (TNF-α) and interleukin-8 (IL-8) for vasculature formation [98]. Interestingly, HMGB1 has also been observed to improve tumor neovascularization by attracting endothelial progenitor cells (EPCs) and mesoangioblasts [99]. EPCs and mesoangioblasts could both differentiate into ECs, and HMGB1 could promote these two cell types to the site of tumors for angiogenesis [100].

#### Apoptosis evasion

Apoptosis is mainly activated through two alternative pathways: the intrinsic pathway triggered by mitochondria and the extrinsic pathway mediated by cell death receptors. Cysteine aspartyl-specific proteases (caspases) that cleave cellular substrates lead to the biochemical and morphological changes of apoptotic cells in both pathways [101]. Crosstalk between HMGB1 and apoptosis has been explored in many cancer cells. Down-regulation of HMGB1 with microRNA 34a, a tumor suppressor gene, leads to inhibition of autophagy and promotes DNA damage in the retinoblastoma cell. Subsequently, the CASP3 and poly (ADP-ribose) polymerase 1 (PARP1) are cleaved, which are important to the apoptotic process. Knockdown of HMGB1 by shRNA similarly induces apoptosis in retinoblastoma cells [102]. Extracellular HMGB1 can protect gastric cancer cells from apoptosis induced by vincristine via transcriptional up-regulation of myeloid cell leukemia-1 (Mcl-1, an anti-apoptotic member of the Bcl-2 protein family). This protective effect can be abolished by knockdown of HMGB1 or inhibition of its release. Additionally, the release of HMGB1 is caused by vincristine-induced cell autophagy, and the up-regulation of Mcl-1 mRNA is mainly through RAGE-mediated signaling, partly through TLR2- and TLR4-mediated signaling [103]. However, several reports have indicated that extracellular HMGB1 is cytotoxic and leads to cell death [104]. Extracellular HMGB1 can induce a special form of cell death in glioblastoma cells, which is accompanied by the formation of vacuolated giant mitochondria and a rapid depletion of mitochondrial DNA (mtDNA). This distinct mode of cell death differs from apoptosis, autophagy, and senescence. The giant mitochondria-associated rhHMGB1-induced cell death is induced by exogenous HMGB1 internalization to mitochondria, independent of TLR2, TLR4, or RAGE signaling [104].

#### Inflammation

The mechanism of tumor progression has been shown to be associated with the local inflammatory reactions, especially chronic persistent inflammation. Extracellular HMGB1 is considered as a proinflammatory cytokine in this inflammation-associated tumorigenesis [88]. Extracellular HMGB1 can promote NFκB transportation to the nucleus and induce expression of inflammatory factor and tumor cell proliferation via TLR4 signaling pathway [105, 106]. HMGB1 is also recognized as an important mediator of p53-dependent hepatic inflammation that exerts a critical pathogenic role in hepatocarcinogenesis. Sustained p53 activation in response to a persistent DNA damage signal, leads to HMGB1 release, which drives pro-tumorigenic liver inflammation. Inhibition of HMGB1 release mitigates carcinogen-induced hepatic injury and tumorigenesis [107]. In addition, extracellular HMGB1 can induce both recruited leukocytes and settled immune cells to release cytokines such as TNF, IL-1 and IL-6, which amplifies and extends the inflammatory response [108].

#### Immunity

Cancer immunity surveillance is an important host defense process to maintain cellular homeostasis. HMGB1 has been characterized as both immune suppression and immune activation properties, which depends on receptors, targeted cells and redox state [54]. HMGB1 has the ability to mature DCs and support the clonal expansion of γ-interferon producing Th1 cells [109]. Immunogenic cell death (ICD) is a cell death modality that stimulates immune response against dead cell antigens. The immunogenic characteristics of ICD are mainly mediated by DAMPs, which include released HMGB1 [110]. In the resting state of diffuse large B-cell lymphoma (DLBCL) cells, HMGB1 co-localized and interacts with STAT3 in the nucleus. Treatment of rituximab induces an inhibition on STAT3 activity and triggers a rapid HMGB1 release and IL-10 reduction in DLBCL patients’ serum. The medium from rituximab-treated DLBCL cells is competent for phagocytosis, dendritic cells maturation, and IFN-γ secretion by cytotoxic T cells, which elicits immune responses [111]. Immune tolerance is essential for a functioning immune system and any approximation of self-nonself discrimination. It is reported that the released HMGB1 from dying cells can be oxidized via caspase dependent production of reactive oxygen species in mitochondria of apoptotic cells, which promotes immunological tolerance [112]. The immune tolerance can be abrogated by blocking oxidation of cysteine resides in HMGB1 at positions C106, suggesting that the oxidation/reduction status of cysteine in HMGB1 is responsible for immunogenicity and tolerance of dying cells [112].

## HMGB1 IN ANTICANCER THERAPY

**Figure 4:**
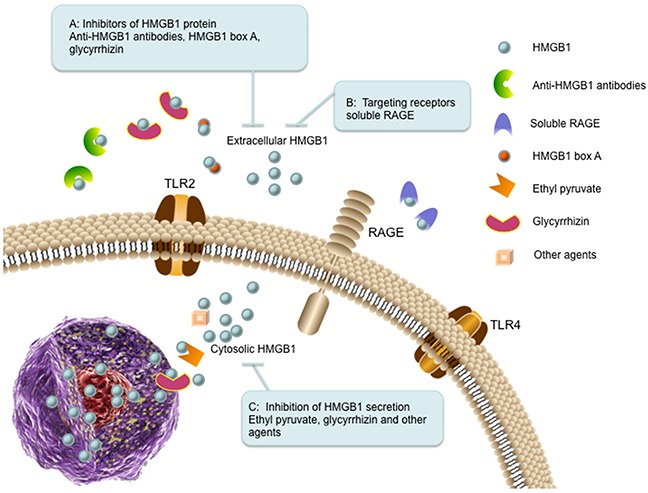
The therapeutic strategies targeting HMGB1 in cancer **(A)** Inhibitors of HMGB1 protein. Extracellular HMGB1 can be blocked by administration of anti-HMGB1 antibodies (binds to HMGB1), HMGB1 box A (antagonizes the B box functional activity of HMGB1), and glycyrrhizin (binds to HMGB1). **(B)** Targeting receptors. Soluble RAGE (sRAGE) acts as a decoy to block HMGB1-RAGE signaling pathway. **(C)** Inhibition of HMGB1 secretion. The secretion of HMGB1 can be inhibited by ethyl pyruvate, glycyrrhizin and other agents (such as quercetin and nicotine).

Currently, several strategies have been proposed to inhibit HMGB1 expression, activity and release in a direct or indirect manner for treatment of cancer as well as various inflammatory diseases (Figure 4).

### Inhibitors of HMGB1 protein

Administration of anti-HMGB1 antibody inhibits liver metastasis of colorectal cancer, suggesting that HMGB1 is a promising target for metastasis inhibition [113]. Anti-HMGB1 neutralizing antibody also significantly decreases the effect of released extracellular HMGB1 from cancer-associated fibroblasts or recombinant HMGB1 on doxorubicin (DOX) resistant breast cancer cells [114]. HMGB1 box A can be utilized as a competitive HMGB1 antagonist and block HMGB1-associated inflammation and cancer [5]. Knockdown of HMGB1 using antisense oligodeoxynucleic acid technology inhibits cancer cell growth and metastasis [18, 115]. Glycyrrhizin (GR), a triterpenoid saponin glucoside of glycyrrhizic acid, can specifically bind to both HMG boxes of HMGB1 and inhibit its chemotactic and mitogenic activities [7, 116]. It has been investigated that GR can protect spinal cord, liver, brain and myocard against ischemia-reperfusion induced injury in animal models by inhibiting HMGB1 cytokine activity [7, 117]. During anticancer therapy, HMGB1 favors to release into the extracellular milieu, which has positive effects on tumor relapse, such as stimulation of cancer cell proliferation, angiogenesis, cell motility and inflammation. As HMGB1 inhibitor, GR impedes HMGB1 induced tumor cell proliferation, migration, blood vessels formation and inflammatory condition [118].

### Targeting receptors

Blockade of HMGB1-RAGE signaling has been observed to suppress tumor growth and metastasis in implanted tumors [92]. Inhibition of the HMGB1-RAGE interaction suppresses activation of MAP kinases, which are the important molecular effector mechanisms linking to tumor progression [92]. Soluble RAGE is an endogenous truncated form of RAGE consisting of the extracellular domain of RAGE, which acts as a decoy to block HMGB1-RAGE signaling pathway in animal tumor models [92, 119].

### Inhibition of HMGB1 secretion

It is well known that monocytes and macrophages can secrete HMGB1 under various stress stimuli [16, 46]. Some cancer cells also have the ability to secrete HMGB1 themselves into the culture media, such as colon cancer and malignant mesothelioma [120, 121]. Current investigations have focused on pharmacological inhibition of HMGB1 secretion. Ethyl pyruvate (EP), a pyruvic acid derivative, is the first described pharmacological inhibitor for HMGB1 secretion. EP impairs HMGB1 secretion by malignant mesothelioma cells and downregulates RAGE expression and NF-κB activation. Moreover, EP reduces HMGB1 serum levels in mice and inhibits the growth of malignant mesothelioma xenografts [122]. As an antioxidant, quercetin reduces circulating level of HMGB1 in animals with established endotoxemia. In macrophage cultures, quercetin inhibits release as well as the cytokine activities of HMGB1 [123]. Glycyrrhizin is also utilized as a HMGB1 secretion inhibitor. It is reported that glycyrrhizin can inhibit HMGB1 phosphorylation and secretion by directly binding to HMGB1 and interacting with two shallow concave surfaces formed by the tow arms of both HMG boxes [124]. Macrophage treatment with nicotine showed prevention of HMGB1 secretion and inhibited activation of NF-κB signaling pathway [125].

## CONCLUSION

HMGB1 is involved in multiple biologic process of cancer, such as tumor growth, tumor cell proliferation, invasion and metastasis. In nucleus, HMGB1 acts as a tumor suppressor via various mechanisms to sustain genome stability, while out of nucleus, it acts as either tumor supporter or tumor suppressor. For instance, extracellular HMGB1 is conducive to ICD-associated antitumor immunity in the early stages of chemotherapy, while it facilitates residual tumor cell survival in the late stages of chemotherapy. Other studies have showed that the redox of HMGB1 plays an important role in tumor cell death and survival. Therefore, the first prerequisite is whether to design a HMGB1 inducer or inhibitor when considering HMGB1 targeted therapeutic, and the targeted treatment of cancer varies at different stages.

Numerous strategies have being employed to restrain the activity and release of HMGB1 in anti-cancer therapeutics. However, there are still flaws in these strategies. It is reported that HMGB1-deficient mice are born alive, but die within 24h due to hypoglycaemia [126]. Inhibition of HMGB1 in nuclear can induce insufficient transcription or DNA repair in normal cells [49]. HMGB1 inhibitor or neutralizing antibody also abates immune response to virus infection and tumor antigen via blocking HMGB1/TLR4 signaling pathway [22]. Accordingly, neutralizing monoclonal antibody of HMGB1 should incorporate the fact that HMGB1/TLR4 signaling modulates inflammatory responses while HMGB1/RAGE signaling promotes tumor growth and metastasis [18, 22]. Moreover, apart from HMGB1, there are several other ligands for RAGE such as S100B and S100P, which are associated with pro-inflammatory activation [127]. Thus, strategies targeting RAGE receptor impair S100B and S100P related functions. The release of HMGB1 is affected by many factors such as cytokines, oxidative stress and cellular stresses. Therefore, interference with HMGB1 release should take all these factors into consideration. Although much attention has been paid on the redox statuses of HMGB1, especially its effect on tumor cell autophagy and apoptosis [128], it is still unknown about how signaling pathways are regulated by different redox forms of HMGB1.

Indeed, HMGB1 has been proved to be a successful therapeutic target in experimental models of diverse infectious and inflammatory diseases [129]. There are no ongoing clinical trials for HMGB1 targeted agents in patients with cancer so far. Further basic and clinical studies are warranted to exploit the explicit structure, location and partners of HMGB1, especially multiple functions of HMGB1 in regulating tumor cell survival and death. Considering the essential role of HMGB1 in innate immunity, methods for local inhibition of HMGB1 need to be invented for the safety of HMGB1 targeting therapy.
